# Access Barriers to Pembrolizumab in Brazil

**DOI:** 10.1001/jamanetworkopen.2025.28585

**Published:** 2025-08-22

**Authors:** Pedro Luiz Serrano Uson Junior, Uelson Donizeti Rocioli, Maria Fernanda Botelho Teixeira, Isabella Sforzin, Guilherme Andriatte, Francisco Tustumi, Fernando Moura, Mitesh Borad, Flavio Tocci Moreira, Carlos Henrique Sartorato Pedrotti

**Affiliations:** 1 Center for Personalized Medicine, Hospital Israelita Albert Einstein, Sao Paulo, Brazil; 2 Department of Oncology, Mayo Clinic, Phoenix, Arizona; 3 Department of Telemedicine, Hospital Israelita Albert Einstein, Sao Paulo, Brazil

## Abstract

This cross-sectional study examines the lawsuits that followed national and international policies for pembrolizumab approval.

## Introduction

The constitutionally guaranteed right to universal health, is a pillar of the public health system in Brazil. However, drugs not approved by National Committee of Incorporation of New Technologies (CONITEC) are not available, even in the setting of ANVISA (Brazilian Health Regulatory Agency) approval. Therefore, patients request them through lawsuits. To assist judges in these demands the Center for Technical Support of the Judiciary (NAT-Jus) was created. It integrates the judicial system with the relevant health institutions, aiming to assist judges in scientific matters. Since 2019, it has released more than 150 000 technical notes based on individual legal demands. Pembrolizumab is an immunotherapy with 36 indications in Brazil (by May 2025).^1^ Not all indications need biomarkers (ie, programmed death-ligand 1 [PD-L1]), making universal approval challenging due to costs.^2^ Considering that pembrolizumab is one of the most legally contested immunotherapies, it is important to quantify legal challenges aligning with national or international guidelines, and how many have favorable outcomes, representing patient access.

## Methods

 This cross-sectional study examines all pembrolizumab technical notes issued by NAT-Jus from January 2019 to March 2024. The study was approved by the Hospital Israelita Albert Einstein ethics committee. The primary objective was to describe how many lawsuits followed national and international policies for pembrolizumab approval, including ANVISA, US Food and Drug Administration (FDA), European Medicines Agency (EMA), and China Food and Drug Administration (CFDA), and the patterns of approvals or rejections. The study followed the Strengthening the Reporting of Observational Studies in Epidemiology (STROBE) reporting guideline. R version 4.5.1 (R Project for Statistical Computing) was used for analyses, which were conducted from February to April 2025. 

## Results

**Figure 1.**
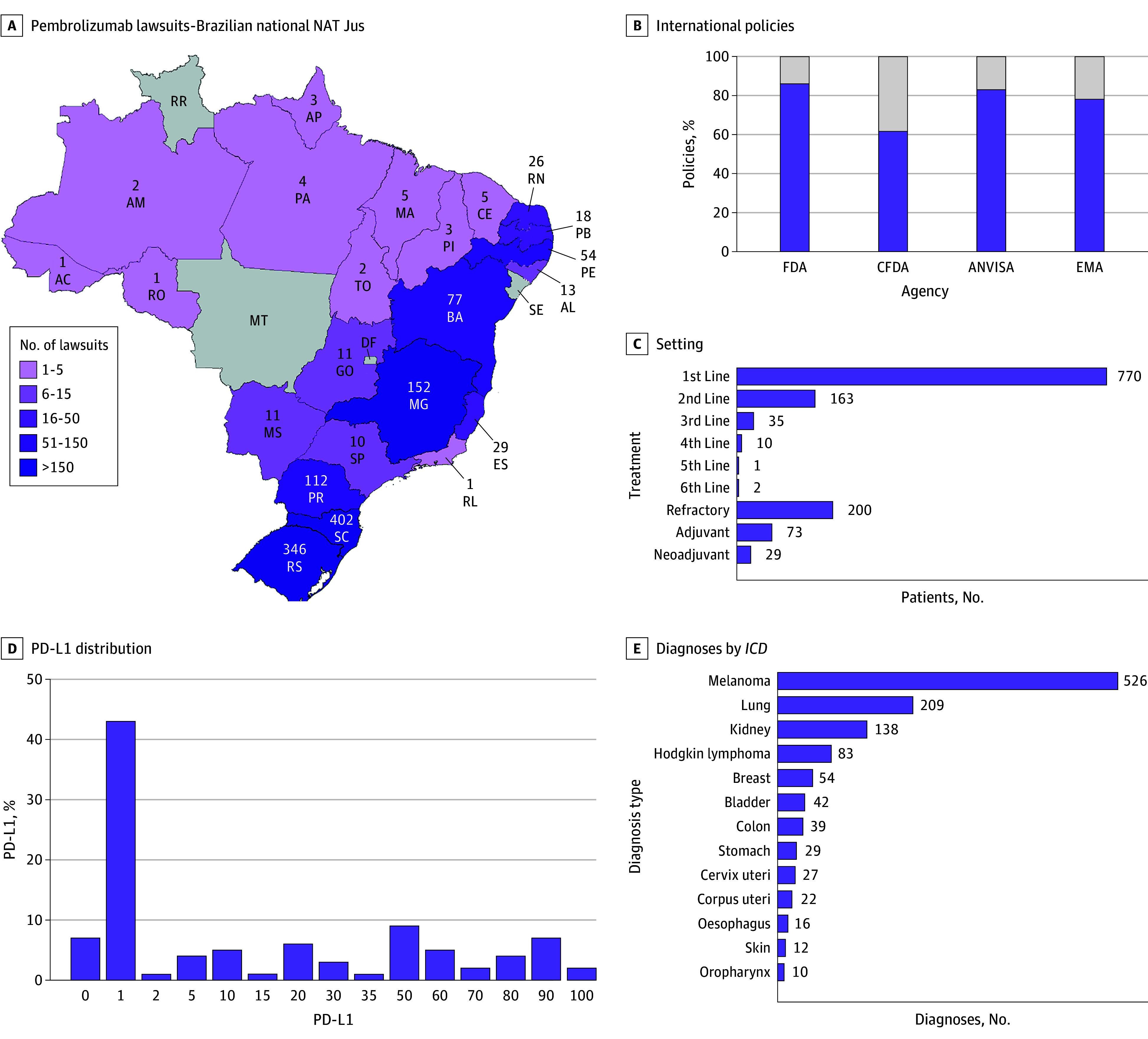
Pembrolizumab Lawsuits Landscape—Brazilian National NAT-Jus Technical Notes ANVISA indicates National Health Surveillance Agency from Brazil; CFDA, China Food and Drug Administration; EMA, European Medicines Agency; FDA, US Food and Drugs Administration; *ICD-10*, *International Statistical Classification of Diseases and Related Health Problems, Tenth Revision*; PD-L1, programmed death-ligand 1.

A total of 1288 pembrolizumab lawsuits were evaluated, 1070 were ANVISA aligned (83%), 1109 were FDA aligned (86%), 1006 were EMA aligned (78%), and 794 based on CFDA approvals (62%). Pembrolizumab lawsuits were for treatment of melanoma (526 [41%]), lung cancer (209 [16%]), renal cancer (138 [11%]), Hodgkin lymphoma (83 [6%]), and breast cancer (54 [4%]) (Figure 1).

**Figure 2.**
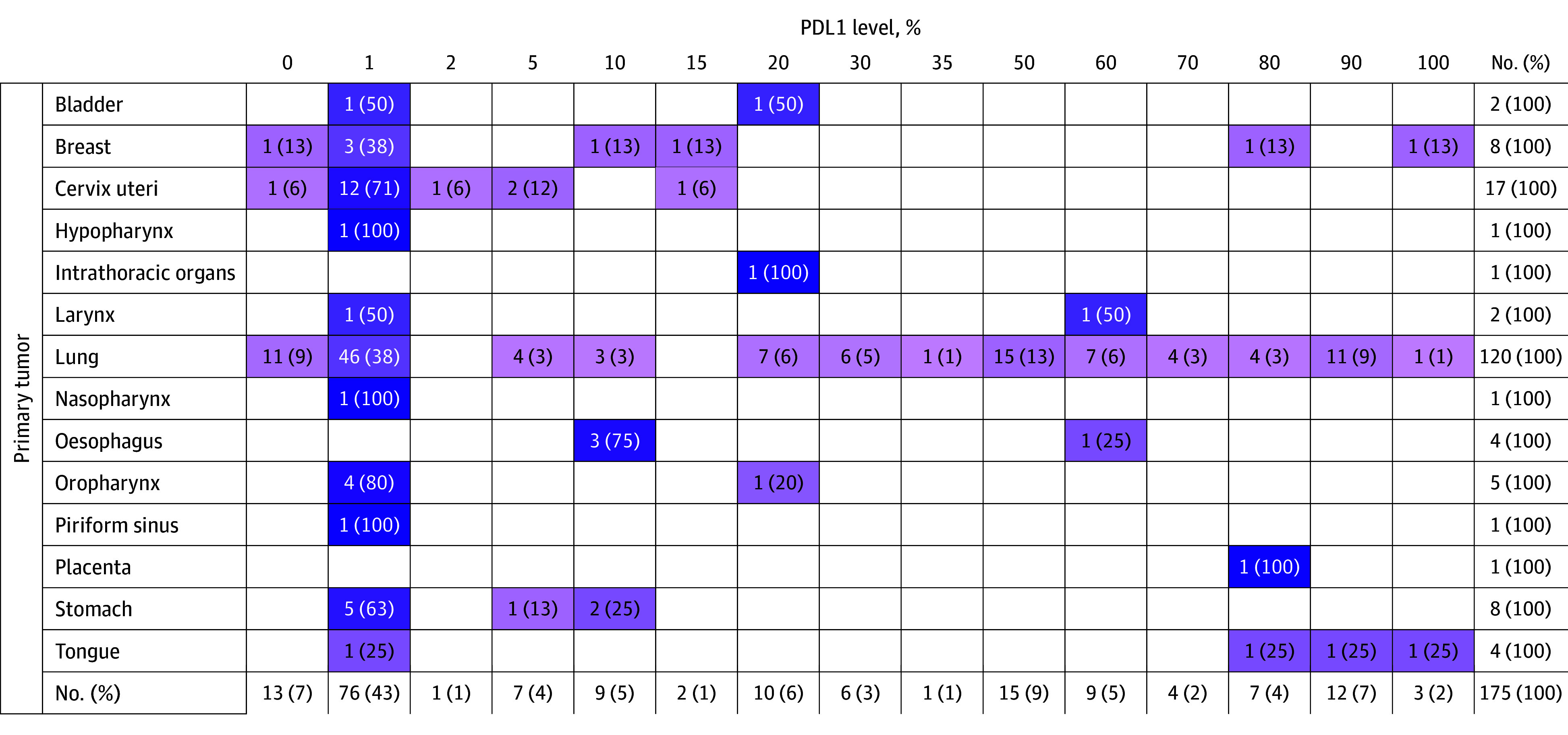
Quantity of Programmed Death-Ligand 1 (PD-L1) Evaluation Across Multiple Tumors This figure illustrates the number of cases that evaluated PD-L1 in each tumor; and the levels of PD-L1 according to each case in pembrolizumab lawsuits.

PD-L1 was included only in 14% of reports (Figure 2). NAT-Jus issued favorable technical notes for 848 (65.8%)—32.8% (423) notes were favorable due to ANVISA approval for the indication, 28.4% (366) were due to CONITEC approval for the indication, and 59 (4.6%) of the approvals were based on positive studies or randomized trials, without CONITEC or ANVISA approvals.

Rejections occurred in 440 cases (34%). Most rejections were due to the absence of examinations in the process (ie, computed tomography scans, magnetic resonance imaging, laboratory), in 281 of 1288 technical notes (22%). Errors of technical indication were the rejection causes in 152 notes (12%). Other justifications (6 [1%]) included indications not evaluated by ANVISA or CONITEC.

## Discussion

Based on 1288 technical notes issued by the NAT-Jus evaluating pembrolizumab, 41% of the notes were associated with melanoma. For patients with metastatic melanoma the indication is approved already by CONITEC, therefore suggesting that the drug is not available. Approximately 60% to 80% of lawsuits were based on national or international approvals (ie, FDA, EMA or CFDA) and mostly based on prospective and/or randomized data. Finally, 33% of the technical notes were emitted as favorable based on ANVISA approval and 28% based on CONITEC approval. A third of the notes were not favorable (34%).

In 2022, Brazilian public health–related expenditure totaled $71.3 billion USD.^3^ Overall, expenditures on health were equivalent to 9.23% of the GDP, lower compared to OECD (Organization for Economic Cooperation and Development) average.^3^ Furthermore, access to available drugs is not easy. In a study that reported access to the drugs in 2013 and 2019, from more than 200 000 people interviewed, the prevalence of full access to medicines were 31.6% and 29.7%, respectively.^4^

On September 26, 2024, the supreme court issued new precedents, CONITEC has now a presumptive force in the access of medication.^5^ Indications like pembrolizumab for microsatellite instable colorectal cancer would be summarily rejected. Other indications that have CONITEC unfavorable recommendation currently include kidney, head and neck, and lung cancer. The direct driver of this position is the concept of cost-effectiveness.^6^ Today, the only recommendation for pembrolizumab was for advanced melanoma in 2020. Based on the data, future approvals of pembrolizumab or other immunotherapies are not likely.

This study has limitations. Technical reports data issued for supporting decision-making in the judiciary presented in this article represent issuances from NAT-Jus Nacional and do not include issuances from other instances, such as state ones.

## Notes

Supplement.
Data Sharing Statement
