# Supplementary material for: Access Barriers to Pembrolizumab in Brazil
Source: JAMA Netw Open. 2025 Aug 22;8(8):e2528585. doi: 10.1001/jamanetworkopen.2025.28585 (PMC12374216; doi:10.1001/jamanetworkopen.2025.28585)
Supplement: Supplement. — Data Sharing Statement [file jamanetwopen-e2528585-s001.pdf]

Data Sharing Statement

Data
Data available: Yes
Data types: Deidentified participant data
How to access data: pedro.serrano@einstein.br
When available: With publication

Supporting Documents
Document types: Other (please specify)
Additional Information: technical notes
How to access documents: pedro.serrano@einstein.br
When available: With publication

Additional Information
Who can access the data: researchers whose proposed use of the data has been approved
Types of analyses: analysis
Mechanisms of data availability: signed data access agreement
